# The efficacy and safety of intrinsic antitachycardia pacing

**DOI:** 10.1002/joa3.13221

**Published:** 2025-01-14

**Authors:** Koumei Onuki, Michio Nagashima, Masato Fukunaga, Keigo Misonou, Maiko Kuroda, Hiroyuki Kono, Tomonori Katsuki, Rei Kuji, Kengo Korai, Kenichi Hiroshima, Kenji Ando

**Affiliations:** ^1^ Department of Cardiology Kokura Memorial Hospital Kitakyushu Japan

**Keywords:** acceleration, implantable cardioverter defibrillator, intrinsic antitachycardia pacing, postpacing interval, ventricular tachycardia

## Abstract

**Background:**

The clinical outcomes of a novel antitachycardia pacing (ATP) algorithm—intrinsic ATP (iATP)—compared to conventional ATP (cATP) have yet to be fully elucidated.

**Methods:**

This retrospective study analyzed 128 patients and 1962 ventricular tachycardia (VT) episodes treated with the iATP or the cATP at Kokura Memorial Hospital. Patients were categorized into two groups: the iATP group (23 patients, 182 episodes) and the cATP group (105 patients, 1780 episodes). We evaluated ATP success rates and baseline patient characteristics on a per‐patient basis. Additionally, we extracted VT that were not terminated by a single ATP and compared ATP success rates using propensity score matching.

**Results:**

Per patient; The iATP group exhibited significantly lower creatinine levels (1.18 ± 0.40 mg/dL vs. 1.82 ± 1.61 mg/dL, *p* = .021) and a shorter follow‐up period (609 ± 323 days vs. 1017 ± 252 days, *p* < .001) compared to the cATP group. ATP success was observed in 19 patients in the iATP group and 62 patients in the cATP group (82.6% vs. 59%, *p* = .054). Per episode; there was no significant difference in ATP success rate (91.8% vs. 92.7%, *p* = .645) or in acceleration rate (1.1% vs. 2.4%, *p* = .274). However, when limited to episodes in which VT was not terminated by a single ATP and propensity score matching was performed, the iATP showed a higher VT termination rate (84.1% vs. 53.6%, *p* < .001) and a lower acceleration rate (0% vs. 10.1%, *p* = .013) than the cATP.

**Conclusions:**

The efficacy and safety of the iATP for VT that was not terminated by the first sequence of ATP was demonstrated.

## Graphical abstract

This study reports the efficacy and safety of intrinsic antitachycardia pacing (iATP) for ventricular tachycardia (VT) not terminated by the first ATP sequence, compared to conventional ATP. In the analysis of Sequence 2 and beyond, the iATP group demonstrated a higher VT termination rate and a lower acceleration rate.

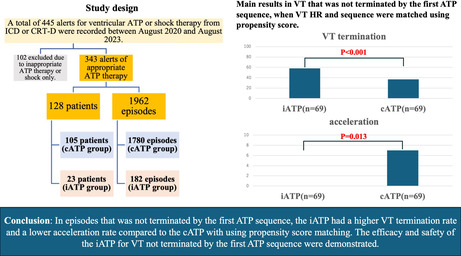

## 1 INTRODUCTION

The implantable cardioverter defibrillator (ICD) offers a well‐proven mortality benefit among patients at high risk of sudden arrhythmic death.^1^ The ICD provides two types of therapy: high‐energy shocks and antitachycardia pacing (ATP). Termination of ventricular tachycardia (VT) with ATP offers a pain‐free alternative to defibrillation shocks. ICD shocks are associated with increased mortality,^2^ making it crucial to improve the success rate of ATP therapy. ATP is primarily categorized into Burst and Ramp therapies, but recently, intrinsic ATP (iATP) has emerged. The iATP is integrated into the Cobalt™ (Medtronic, Minneapolis, USA) and utilizes a novel automated ventricular ATP algorithm that uses the postpacing interval (PPI) to design subsequent ATP sequences based on the analysis of prior failed ATP sequences. Specifically, when the initial ATP sequence fails to terminate VT, the iATP makes two modifications. First, the iATP calculates the number of S1 pulses required to entrain the VT circuit using the postpacing interval (PPI). Second, the device decreases the S2 pulse interval until it terminates the VT. This mechanism facilitates access to the excitable gap of VT, and superior clinical outcomes are anticipated compared to conventional ATP (cATP), especially in terminating VT that does not cease with a single sequence.

Computational models have demonstrated the efficacy of automated ATP (AATP) for VT that is refractory to Burst therapy.^3^ In this study, the AATP exhibited a significantly higher VT termination rate than Burst therapy without increasing the acceleration rate. Furthermore, with each additional sequence, the difference in termination rates between AATP and Burst therapy became more pronounced. However, this study is based on computer models, and this AATP algorithm differs from that of the iATP. There are few reports on the real‐world clinical data of the iATP. Kamakura T et al. reported that the iATP was effective for VT that could not be terminated by Burst therapy,^4^ suggesting that the iATP could be effective against cATP‐resistant VT. Similarly, several case reports have demonstrated the utility of the iATP in scenarios where the cATP was ineffective,^5^, ^6^, ^7^, ^8^ but the situations in which the iATP should be employed and the patient populations for which it would be effective remain unclear.

Therefore, we aimed to investigate the efficacy and safety of the iATP for VT in comparison to the cATP, with a particular focus on analyzing the utility of the iATP for VT that is not terminated by the initial sequence of therapy.

## 2 METHODS

### 2.1 Study population

This study was a retrospective, observational analysis conducted at Kokura Memorial Hospital, Kitakyushu, Japan. We included patients who underwent de novo ICD or Cardiac Resynchronization Therapy‐Defibrillator (CRT‐D) implantation at our hospital and received appropriate ATP therapy for ventricular arrhythmia between August 2020 and August 2023.

Patients were excluded if they (1) had Cobalt™ devices with settings intentionally changed from the iATP to the cATP by the operator, (2) lacked detailed records of ATP episodes, or (3) received manual ATP during ablation procedures, in the emergency department, or in similar situations. Subsequently, patients were categorized into two groups: an iATP group and a cATP group. We evaluated the VT termination rate and acceleration rate per episode, as well as baseline clinical characteristics for each patient.

The research protocol received approval from the ethics committee at Kokura Memorial Hospital and adhered to the principles outlined in the Declaration of Helsinki. Written informed consent was waived because of the retrospective study design. This study was registered with http:// www.umin.ac.jp, unique identifier UMIN000054126.

### 2.2 Device programing

The ICD/CRT‐D programming, including ATP modes such as intervals for VT detection, burst therapy, ramp therapy, or the iATP pacing mode, pacing sequence number, percentage shortening of ATP as a reference to VT cycle length, and the settings for Fast VT and ventricular fibrillation (VF) zones, were not predetermined and were selected and programmed at each physician's discretion based on their patients' clinical and electrophysiological backgrounds. However, in all patients, at least one train of ATP was programmed for the VT, Fast VT, or VF zone before shock. The study includes cases where ATP was performed before or during charging, prior to shock delivery in the VF zone. When configuring the iATP with Cobalt™, it was set to 3–8 iATP sequences at first in every case, followed by either burst therapy, ramp therapy, or both settings. No patients had the cATP programmed before the iATP. In models other than Cobalt™, burst therapy at 88% of the VT cycle length was usually set first, followed by ramp pacing in subsequent sequences, except for some exceptions individually determined by the physicians.

### 2.3 Data collection and follow‐up

Clinical follow‐up data were systematically gathered from medical records or through telephone contact with patients, their families, or referring physicians. For patients with a history of multiple ATP therapies, clinical data (including medication, ECG findings, and whether VT ablation was performed) were recorded based on the most recent appropriate ATP therapy. The underlying conditions were classified into Ischemic Cardiomyopathy (ICM), Dilated Cardiomyopathy (DCM), Hypertrophic Cardiomyopathy (HCM), Cardiac Sarcoidosis, Cardiac Amyloidosis, and Others. The follow‐up period for patients who had the device implanted before August 1, 2020, began on August 1, 2020, and for those implanted after this date, it began on the day of surgery. The end of the follow‐up period was defined as the day the follow‐up was discontinued, the day of death, or August 31, 2023, if neither of the former conditions applied.

### 2.4 Study outcomes and definitions

The primary outcome was the ATP success rate for VT. Success per episode was defined as VT termination without the need for shock therapy, while success per patient was defined as all episodes with successful termination by ATP therapy. The secondary outcomes were (1) all‐cause mortality per patient and (2) acceleration rate per episode.

Additionally, comparisons were made for ATP episodes with two or more sequences. There are two reasons for this approach: first, there is no significant difference in programming between the iATP and cATP when termination occurs in the first sequence; second, these comparisons include one‐shot ATP performed before a shock in the VF zone. Furthermore, propensity score matching was used to adjust for VT cycle length and the number of sequences between the two groups.

### 2.5 Statistical analysis

The data are presented as mean ± standard deviation for continuous variables and number (percentage) for categorical variables. The homogeneity of baseline characteristics was assessed by means of t‐test and chi‐square test for continuous and categorical variables, respectively. We used propensity score matching to adjust the background information (VT heart rate and the number of sequences) between the iATP group and the cATP group. The standardized mean difference (SMD) was used to evaluate the difference in background information after propensity score matching. Statistical analyses for the study were conducted utilizing R software version 3.5.2 (R Foundation for Statistical Computing, Vienna, Austria). A two‐sided *p*‐value of less than .05 was deemed to indicate statistical significance.

## 3 RESULTS

### 3.1 Study population

**FIGURE 1:**
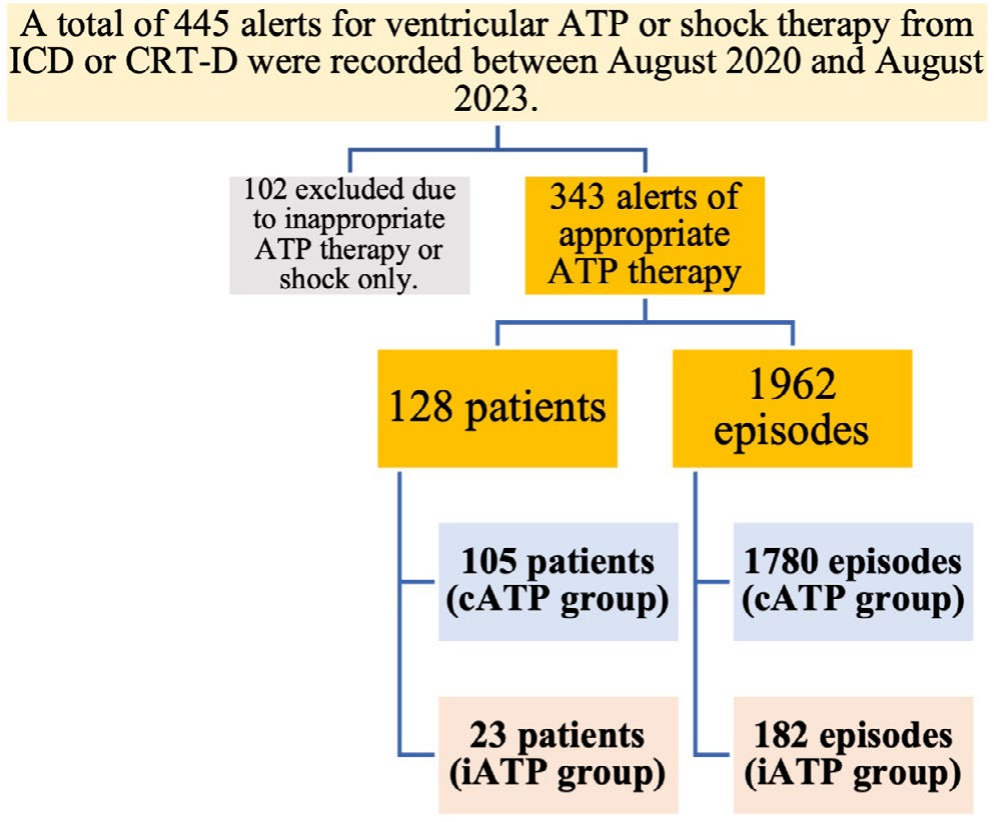
Study flowchart. CRT‐D, cardiac resynchronization therapy‐defibrillator; ICD; implantable cardioverter defibrillator. Other abbreviations as in Table 1. In patients underwent de novo ICD or CRT‐D implantation in our hospital, we analyzed the patients who had appropriate ATP therapy for ventricular arrhythmia between August 2020 and August 2023. A total of 445 alerts for ventricular ATP or shock therapy from ICD or CRT‐D were recorded. Finally, the total 128 patients and 1962 episodes were enrolled.

A total of 445 alerts for ventricular ATP or shock therapy from ICD or CRT‐D were recorded between August 2020 and August 2023. Ultimately, 128 patients and 1962 episodes were included in the current study (Figure 1). These patients and episodes were divided into the iATP group (23 patients, 182 episodes) and the cATP group (105 patients, 1780 episodes). One patient in each group was lost to follow‐up due to relocation. No patients changed their device settings from iATP to cATP during the follow‐up period. Remote monitoring follow‐up was conducted in 78.3% of the iATP group and 65.7% of the cATP group (*p* = .326). Within the cATP group, 7 patients had Ramp therapy preceding Burst therapy, and 4 had Scan set before Burst therapy. All other patients were initially set to Burst therapy, and if ATP did not terminate the VT, shock therapy was administered.

### 3.2 Patient baseline clinical characteristics

The baseline clinical characteristics are summarized in Table 1. The iATP group had significantly lower serum creatinine levels (1.18 ± 0.40 mg/dL vs. 1.82 ± 1.61 mg/dL, *p* = .021) and a shorter follow‐up period (609 ± 323 days vs. 1017 ± 252 days, *p* < .001) compared to the cATP group. Left ventricular ejection fraction (LVEF) was 37.72 ± 16.43% in the iATP group and 33.20 ± 16.25% in the cATP group (*p* = .160). By the end of the follow‐up period, VT ablation had been performed in 4 patients (17.4%) in the iATP group and 39 patients (37.1%) in the cATP group (*p* = .089). There were 4 patients in the iATP group and 32 patients in the cATP group experiencing VT storm, defined as having three or more sustained episodes of VT within a 24‐hour period. ATP success was observed in 19 patients in the iATP group and 62 patients in the cATP group (82.6% vs. 59%, *p* = .054). No significant differences were found in age, sex, underlying conditions, ratio of primary to secondary prevention, device type (ICD or CRT‐D), medication history, electrocardiogram findings, all‐cause mortality, cardiac deaths, or arrhythmia‐related deaths.

**TABLE 1:** Patient baseline clinical characteristics.

	cATP (*n* = 105)	iATP (*n* = 23)	*p* value
Age	68.95 ± 11.76	73.52 ± 10.31	.147
Male sex	85 (81.0%)	19 (82.6%)	1
Follow‐up period (day)	**1017.47 ± 252.38**	**608.61 ± 322.73**	**<.001**
Remote monitoring	69 (65.7%)	18 (78.3%)	.326
Cr	**1.82 ± 1.61**	**1.18 ± 0.40**	.**021**
LVEF	33.20 ± 16.25	37.72 ± 16.43	.16
History of VT ablation	39 (37.1%)	4 (17.4%)	.089
VT strom	13 (12.4%)	3 (13.0%)	.931
HT	68 (64.8%)	19 (82.6%)	.138
DLp	59 (56.2%)	13 (56.5%)	1
DM	34 (32.4%)	8 (34.8%)	.811
Disease			
ICM	42 (40.0%)	9 (39.1%)	.923
DCM	33 (31.4%)	6 (26.1%)	
HCM	8 (7.6%)	2 (8.7%)	
Sarcoidosis	11 (10.5%)	4 (17.4%)	
Amyloidosis	2 (1.9%)	0 (0.0%)	
OtherDisease	9 (8.6%)	2 (8.7%)	
Medication			
TypeIII antiarrhythmic	62 (60.2%)	11 (47.8%)	.351
*β* blocker	98 (93.3%)	20 (87.0%)	.384
Diuretics	77 (73.3%)	14 (60.9%)	.309
Statin	56 (53.3%)	11 (47.8%)	.652
Nitrate	9 (8.6%)	1 (4.3%)	.689
Cardiac glycosides	26 (24.8%)	3 (13.0%)	.281
Ace/ARB/ARNi	84 (80.0%)	18 (78.3%)	.783
SGLT2R‐i	52 (49.5%)	10 (43.5%)	.65
MRA	71 (67.6%)	16 (69.6%)	1
ECG			
AF rhythm	15 (14.3%)	3 (13.0%)	.895
Heart rate	67.90 ± 11.54	65.04 ± 8.05	.263
PR	153.66 ± 89.19	184.43 ± 82.15	.131
QRS duration	136.21 ± 29.75	125.83 ± 37.75	.152
QTc	465.99 ± 49.48	448.74 ± 50.64	.134
Device type			
ICD	58 (55.2%)	16 (69.6%)	.249
CRT‐D	47 (44.8%)	7 (30.4%)	
Primary prevention	68 (64.8%)	15 (65.2%)	1
Secondary prevention	37 (35.2%)	8 (34.8%)	
ATP success	62 (59.0%)	19 (82.6%)	.054
All‐cause mortality	23 (21.9%)	4 (17.4%)	.782
Cardiac deaths	15 (14.3%)	3 (13.0%)	.895
Arrhythmia related deaths	3 (2.9%)	0 (0%)	1

*Note*: Values are expressed as mean ± standard deviation or frequency count and percentages. The homogeneity of baseline characteristics was assessed by means of *t*‐test and chi‐square test for continuous and categorical variables, respectively. A two‐sided *p*‐value of less than .05 was deemed to indicate statistical significance. Bold vlaues indicate statistically significance.

Abbreviations: Ace, angiotensin‐converting enzyme inhibitor; AF, atrial fibrillation; ARB, angiotensin II receptor blocker; ARNi, angiotensin receptor‐neprilysin inhibitor; ATP, antitachycardia pacing; cATP, conventional antitachycardia pacing; Cr, Creatinine; DCM, dilated cardiomyopathy; DLp, dyslipidemia; DM, diabetes mellitus; ECG, electrocardiogram; HCM, hypertrophic cardiomyopathy; HT, hypertension; iATP, intrinsic antitachycardia pacing; ICM, ischemic cardiomyopathy; LVEF, left ventricular ejection fraction; MRA, mineralocorticoid receptor antagonist; SGLT2R‐I, sodium‐glucose co‐transporter 2 inhibitor; VT, ventricular tachycardia.

### 3.3 Episode characteristics and results

**FIGURE 2:**
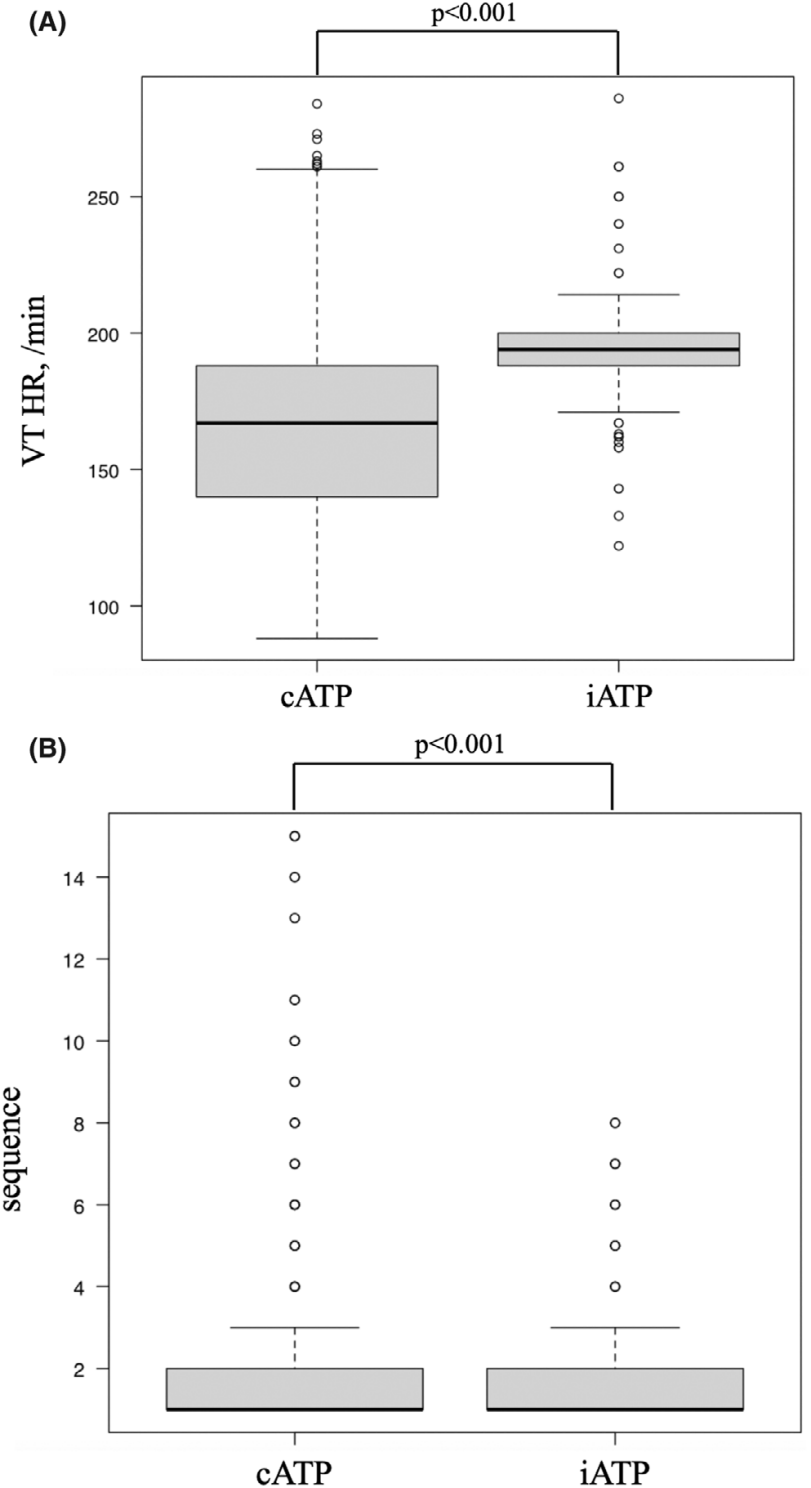
Box plot of VT HR (A) and number of sequence (B) in the iATP group and the cATP group. Abbreviations as in Tables 1 and 2. In the iATP group, the mean VT heart rate was higher (median, 194 bpm vs. 167 bpm, *p* < .001), and the number of sequences was fewer (mean ± standard deviation, 1.79 ± 1.91 vs. 2.10 ± 1.85, *p* < .001) than in the cATP group.

The comparison results for all episodes are shown in Table 2. The number of ATP therapeutic episodes was 182 for the iATP group and 1780 for the cATP group. Between the two groups, there was no significant difference in ATP success rates (91.8% vs. 92.7%, *p* = .645) or in acceleration rates (1.1% vs. 2.4%, *p* = .274). The episodes in which VTs were terminated by the first ATP sequence were 107 episodes in the iATP group and 1261 episodes in the cATP group (58.8% vs. 70.1%, *p* = .001). In the iATP group, the mean VT heart rate was higher (median, 194 bpm vs. 167 bpm, *p* < .001), and the number of sequences was higher (mean ± standard deviation, 2.10 ± 1.85 vs. 1.79 ± 1.91, *p* < .001) than in the cATP group (Figure 2). The results when VT heart rate and the number of sequences were matched using propensity score are shown in Table 3. A total of 161 episodes were selected from both the iATP group and the cATP group. The iATP showed a higher success rate (95.7% vs. 85.7%, *p* = .003). The termination rate at the first ATP sequence did not differ between the two groups (34.2% vs. 33.5%, *p* = 1). After propensity score matching, there was no significant difference in the number of sequences (1.77 ± 1.46 vs. 1.77 ± 1.46, *p* = 1, SMD <0.001), but VT HR was higher in the cATP group (median, 194 bpm vs. 194 bpm, mean ± standard deviation, 190.04 ± 17.28 vs. 193.06 ± 16.39, *p* = .019, SMD = 0.157). When limited to episodes that VT was not terminated in the first ATP sequence, propensity score matching was similarly used to select 69 episodes from both the iATP and cATP groups. There was no significant difference in VT heart rate (median, 194 bpm vs. 194 bpm, *p* = .243, SMD = 0.157) and the number of sequences (mean ± standard deviation, 3.81 ± 1.97 vs. 3.77 ± 2.09, *p* = .817, SMD = 0.021) between the two groups. The matched iATP group demonstrated a higher ATP success rate (84.1% vs. 53.6%, *p* < .001) and a lower acceleration rate (0% vs. 10.1%, *p* = .013). There were 15 episodes that were not terminated by the iATP sequence, and all of them failed to terminate with the followed programmed cATP sequence, resulting in shock therapy.

**TABLE 2:** Episode characteristics between iATP group and cATP group.

	cATP (*n* = 1780)	iATP (*n* = 182)	*p*‐value
Success	1650 (92.7%)	167 (91.8%)	0.645
Failure	130 (7.3%)	15 (8.2%)	
Termination by 1st sequence	**1261 (70.1%)**	**107 (58.8%)**	**0.001**
Accelerate	42 (2.4%)	2 (1.1%)	0.274
Sequence	**1.79 ± 1.91**	**2.10 ± 1.85**	**<0.001**
VT HR	**167 (144, 188)**	**194 (188, 200)**	**<0.001**

*Note*: Values are median (lower and upper qualities), mean ± standard deviation or number (percentage). A two‐sided *p*‐value of less than .05 was deemed to indicate statistical significance. Other abbreviations were similar to that of Table 1. Bold vlaues indicate statistically significance.

Abbreviation: VT HR, mean heart rate of ventricular tachycardia.

**TABLE 3:** Episode characteristics when VT HR and sequence were matched using propensity score.

	All episodes				2nd or later			
	cATP (*n* = 161)	iATP (*n* = 161)	*p*‐value	SMD	cATP (*n* = 69)	iATP (*n* = 69)	*p*‐value	SMD
Success	**138 (85.7%)**	**154 (95.7%)**	.**003**	0.347	**37 (53.6%)**	**58 (84.1%)**	**<.001**	0.696
Failure	23 (14.3%)	7 (4.3%)			32 (46.4%)	11 (15.9%)		
Termination by 1st sequence	54 (33.5%)	55 (34.2%)	1	0.013				
Accelerate	7 (4.3%)	1 (0.6%)	.067	0.241	**7 (10.1%)**	**0 (0%)**	.**013**	0.475
Sequence	1.77 ± 1.46	1.77 ± 1.46	1	<0.001	3.81 ± 1.97	3.77 ± 2.09	.817	0.021
VT HR	**194 (188, 200)**	**194 (182, 200)**	.**019**	0.183	194 (188, 200)	194 (188, 200)	.243	0.157

*Note*: Values are median (lower and upper qualities), mean ± standard deviation or number (percentage). For sequence and mean heart rate of ventricular tachycardia (VT HR) were shown after adjustment by propensity score matching. The standardized mean difference (SMD) was indicated on the right side. A two‐sided *p*‐value of less than .05 was deemed to indicate statistical significance. Other abbreviations were similar to that of Tables 1 and 2. Bold vlaues indicate statistically significance.

## 4 DISCUSSION

### 4.1 Efficacy of the iATP

ATP therapy uses low‐energy patterned stimulation to terminate VT; it has programmable options and is both painless and effective in monomorphic VT. However, ATP may also accelerate tachycardia or cause degeneration into VF.^9^ There is ongoing debate about whether ATP should be actively used for VT, as well as about the appropriate number of ATP therapies and the tachycardia zone settings.

The iATP is a novel automated ventricular ATP algorithm that designs the next ATP sequence based on the analysis of the prior failed ATP sequence. While the cATP requires individualized settings for each patient, with adjustments needed whenever VT characteristics change, the iATP automatically adjusts pulse count and timing, offering potential benefits in acute‐phase VT treatment when medical intervention may not be feasible. However, the clinical data on the iATP remain insufficient, and its effectiveness is still uncertain.

The main findings of the current study were as follows: (1) in the per‐patient analysis, the iATP tended to be superior to the cATP in terms of the VT termination rate, (2) in the per‐episode analysis, there was no significant difference in VT termination rate and acceleration rate when compared overall, (3) when VT heart rate and the number of sequences were matched using propensity score, the iATP showed a higher success rate than the cATP (95.7% vs. 85.7%, *p* = .003), but the termination rate at the first ATP sequence did not differ between the two groups (34.2% vs. 33.5%, *p* = 1). (4) when limited to episodes in which VT was not terminated in the first ATP sequence and propensity score matching was performed, the iATP showed a higher VT termination rate (84.1% vs. 53.6%, *p* < .001) and a lower acceleration rate (0% vs. 10.1%, *p* = .013) than the cATP. To the best of our knowledge, the current study is the first to demonstrate the efficacy and safety of the iATP for VTs that were not terminated by the first ATP sequence. In the overall episodes analysis, the cATP appeared to have a higher termination rate in the first sequence (iATP 58.8% vs. cATP 70.1%, *p* = .001); however, analysis using propensity score matching revealed that the termination rates were equivalent (34.2% vs. 33.5%, *p* = 1). This result is reasonable considering the algorithms of iATP and cATP.

In the overall per‐patient analysis, ATP success was observed in 19 patients in the iATP group and 62 patients in the cATP group (82.6% vs. 59%, *p* = .054). Although this difference was not statistically significant, the iATP tended to be superior to the cATP in terms of VT termination rate. One reason for the lack of significance is that many episodes were terminated by the first ATP sequence (Table 2). Then, we conducted an additional analysis of all 551 episodes that were not terminated by the first sequence (Table 4). The episodes were divided into a success group (*n* = 449), where VT was terminated after the second sequence or later, and a failure group (*n* = 102), where VT was not terminated. In the success group, the VT heart rate was lower (median, 162 bpm vs. 182 bpm, *p* < .001), and the number of sequences was smaller (3.47 ± 2.11 vs. 5.89 ± 3.52, *p* < .001), but the proportion of the iATP did not differ (13.4% vs. 10.8%, *p* = .623). In the episodes that were not terminated by the first sequence, multivariate logistic regression analysis was performed to predict the factors contributing to subsequent ATP success. The use of iATP (Odds ratio = 6.160, 95% CI: 2.640–14.400, *p* < .001), low VT heart rate (Odds ratio = 0.954, 95% CI: 0.942–0.965, *p* < .001), and a small number of sequences (Odds ratio = 0.669, 95% CI: 0.607–0.738, *p* < .001) were statistically associated with an increased ATP success rate. In other words, the use of iATP remained statistically significant for ATP success in the logistic regression analysis, even after adjusting for VT heart rate and the number of sequences.

**TABLE 4:** Episode characteristics that were not terminated by the first ATP.

	Success (*n* = 449)	Failure (*n* = 102)	*p*‐value
iATP	60 (13.4%)	11 (10.8%)	.623
VT HR	**162 (128,181)**	**182 (158,197)**	**<.001**
Sequence	**3.47 ± 2.11**	**5.89 ± 3.52**	**<.001**

*Note*: Values are median (lower and upper qualities), mean ± standard deviation or number (percentage). A two‐sided *p*‐value of less than .05 was deemed to indicate statistical significance. Abbreviations: CI; Confidence Interval. Other abbreviations were similar to that of Tables 1 and 3. Bold vlaues indicate statistically significance.

In addition, we also conducted a univariate logistic regression analysis of predictors for successful ATP therapy for the three factors that showed differences in patient characteristics between the two groups (Table 5). The results suggested that the cATP group, which had higher serum creatinine levels (Odds ratio = 0.775, 95% CI: 0.592–1.01, *p* = .062) and a higher incidence of prior VT ablation (Odds ratio = 0.462, 95% CI: 0.217–0.982, *p* = .045), may have included relatively more severe patients who experienced a higher frequency of VT episodes requiring ATP therapy. Additionally, since the cATP group had a longer device implantation period, there is concern that the progression of cardiomyopathy may lead to an increase in scar tissue, which could result in a higher incidence of VT.

**TABLE 5:** Univariate logistic regression analysis of predictors for successful ATP therapy for the three factors that showed differences in patient characteristics between the iATP group and the cATP group.

	Odds ratio	95% CI	*p*‐value
Cr	0.775	0.592–1.01	0.062
follow‐up period	1	0.998–1.00	0.545
VT ablation	**0.462**	**0.217–0.982**	**0.0449**

*Note*: When the dependent variable was continuous, multiple regression analysis was performed, and when it was binary, logistic regression analysis was conducted. A two‐sided *p*‐value of less than .05 was deemed to indicate statistical significance. Abbreviations were similar to that of Tables 1, 2, 3, 4. Bold vlaues indicate statistically significance.

Although there is not yet large‐scale clinical trial data on the efficacy of the iATP, several reports exist for its predecessor model, AATP. The feasibility and safety of the AATP were tested in a multicenter, prospective, single‐cohort study involving 144 ambulatory patients.^10^ In total, 669 sustained monomorphic VTs from 49 patients were adjudicated, with an overall termination rate of 80.1%. In a report of real‐world data from the United States, Australia, Canada, and New Zealand, the success rate of AATP was 78% for all episodes after adjustment with Generalized Estimating Equations (GEE).^11^ There were 164 cases (7%) where AATP failed, and shock therapy was administered. Of all successful AATP cases, 77% were terminated during the first ATP sequence. These data are comparable to the figures in the current study.

Yanagisawa et al. reported the benefit of the iATP as a secondary therapy after the failure of conventional ATP to terminate VT.^12^ In this report, the effectiveness was compared between a group with the cATP programmed first followed by the iATP and a group with the iATP programmed after the cATP. In the current study, however, we compared a group with the iATP programmed from the first sequence to a group with only the cATP. The iATP did not increase acceleration even in sequences of 2 or more. And in 15 episodes where VT was not terminated by the iATP, subsequent cATP also failed to terminate the episodes. Therefore, it may be effective to program the iATP from the first sequence.

Applying the iATP to all patients with ICD implants might be excessive, but there is no reliable way to predict responsiveness to ATP or the future occurrence of VT/VF, as the characteristics of VT and patient backgrounds can change over time. The advantage of the iATP is that it can automatically adjust the ATP settings if the arrhythmia is not terminated in one ATP therapy, without the need for in‐person reprogramming. Particularly in facilities where VT ablation cannot be performed immediately or remote monitoring is not available, a reduction in shock therapy through ATP may be expected for patients experiencing VT storm. Further prospective studies are needed to determine which patients are most suitable for the iATP.

### 4.2 Acceleration rate and effect of VT rate

There have been no data to date indicating that the acceleration rate of the iATP is higher compared to the cATP; however, concerns have been raised about a potential increase in the acceleration rate due to the application of several S1 pulses followed by S2 and S3 pulses to achieve a shorter pacing cycle length. The current study found that the iATP had a significantly lower acceleration rate compared to the cATP when limited to episodes in which VT was not terminated in the first ATP sequence, using propensity score matching (Table 3). This is thought to be because the iATP calculates the number of S1 pulses and adjusts to entrain the VT circuit with the minimum number of pulses. However, caution is needed, as the occurrence of acceleration in this study was less frequent compared to past reports.^3^, ^10^ In our hospital, episodes with rates >200/min were categorized in the VF zone, which may be related to the fact that only two iATP sequences were performed. It remains unclear whether increasing the number of iATP sequences would improve the VT termination rate.

**FIGURE 3:**
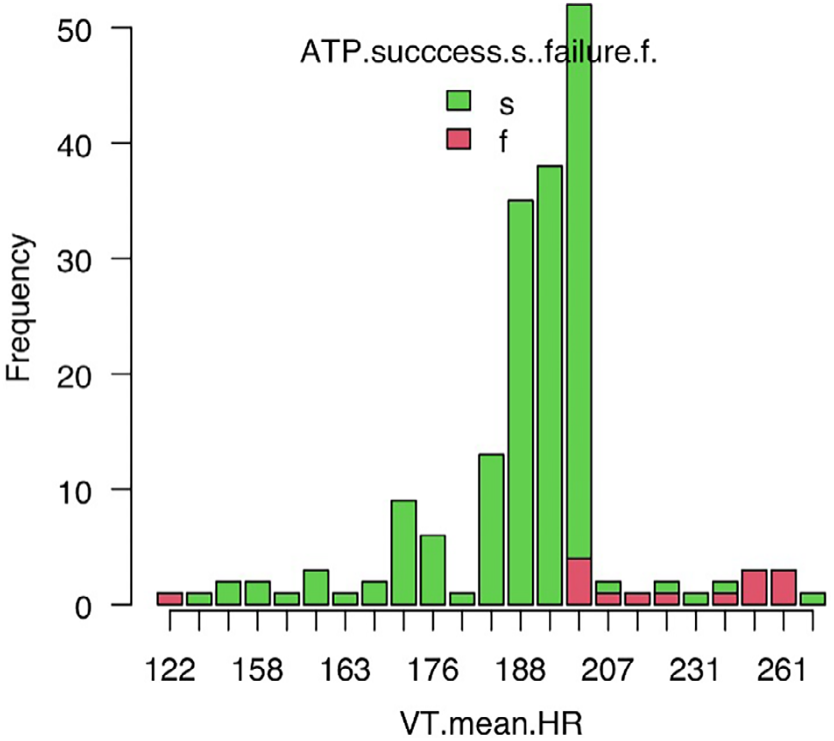
VT mean HR for each episode of the iATP. Abbreviations as in Tables 1 and 2. Most iATP failure occurred in relatively fast rate VT that was over 200/min (shorter than 300 ms).

Regarding the cATP, Awad et al. reported that a short VT cycle length (shorter than 310 ms), a high number of burst therapies, a short burst adaptive cycle length, and the use of scan and ramp therapy are all predictors of VT acceleration.^13^ Thus, it is known that in fast VT, not only does the VT termination rate decrease with ATP, but the acceleration rate also increases. The same might be true for the iATP as well. In the current study, most iATP failures occurred in relatively fast rate VT, with rates over 200/min (shorter than 300 ms) (Figure 3). Similarly, in past reports, when episodes were divided by VT cycle length, those with <320 ms had a success rate of 75%, while those with ≥320 ms had a success rate of 80%.^11^ Although there are no reports on the acceleration rate of the iATP, the acceleration rate of AATP was reported to be 4.2% for fast VT and 1.3% for slower VT.^3^ This is believed to be because entering the excitable gap becomes more challenging in fast VT.

### 4.3 Device programming

ATP provides painless treatment for patients with VT and may conserve battery life if the shock therapy is avoided. Angel A et al. reported that by utilizing long detection intervals and ATP, the number of shock therapies in ICD patients could be reduced.^14^ In the current study, the efficacy and safety of iATP for VTs that were not terminated by the first ATP sequence were demonstrated, suggesting that iATP may reduce the need for shock treatments. Notably, there were 15 episodes that were not terminated by the iATP sequence, and all of them failed to terminate with the followed programmed cATP sequence, resulting in shock therapy. Therefore, we propose setting the iATP from the first sequence in VT treatment with ICD. However, it is crucial not to delay necessary shock therapy. For fast VT or those that cause loss of consciousness, it may be beneficial to use fewer iATP sequences by employing the VF zone or Fast VT zone. Conversely, for VTs with lower heart rates, increasing the number of iATP sequences might be considered due to the higher likelihood of successful termination. It is essential to determine the usefulness of ATP after considering various patient factors, such as the risk of VT, underlying conditions, and patient prognosis.

### 4.4 Limitations

The present study has several limitations. First, this study was retrospective; therefore, the sample size could not be calculated. Although we performed propensity score matching to account for variations in baseline clinical characteristics between the iATP and cATP groups, the inherent potential for bias in this study is inevitable, which might influence the conclusions drawn. Second, the selection of the ATP system was left to the operator's discretion. NID (number of intervals to detect) and Fast VT zone settings were individually configured for each patient, resulting in a lack of uniformity in the device settings. Third, the iATP was a relatively new therapy, and the follow‐up period for the iATP group was shorter than that of the cATP group. Patients who experienced at least one episode of ATP failure were classified into the Failure group. Therefore, in the cATP group with a longer observation period, there is a possibility that more failures may have been observed when compared on a per‐patient basis. In addition, VTs that naturally resolved before ATP completion might have been included in the success group. Finally, since adjustment with GEE was not performed, the treatment outcomes of patients with VT storm may have influenced the overall study results.

## 5 CONCLUSIONS

In the per‐patient analysis, the iATP tended to be superior to the cATP in terms of the VT termination rate. In the per‐episode analysis, there was no significant difference in VT termination rate and acceleration rate overall. In episodes that was not terminated by the first ATP sequence, the iATP had a higher VT termination rate and a lower acceleration rate compared to the cATP with using propensity score matching. The efficacy and safety of the iATP for VTs not terminated by the first ATP sequence were demonstrated. Setting the iATP from the first sequence in VT treatment with ICD is considered reasonable in terms of safety and efficacy.

## FUNDING INFORMATION

None.

## CONFLICT OF INTEREST STATEMENT

All authors have no relevant financial or nonfinancial interests to disclose.

## ETHICS STATEMENT

The research protocol received approval from the ethics committee at Kokura Memorial Hospital and adhered to the principles outlined in the Declaration of Helsinki.

## INFORMED CONSENT

Not applicable (Written informed consent was waived because of the retrospective study design).

## CLINICAL TRIAL REGISTRATION

This study was registered with http://www.umin.ac.jp, unique identifier UMIN000054126.

## ANIMAL STUDIES

N/A.

## Data Availability

The data that support the findings of this study are available from the corresponding author upon reasonable request.
